# Musculoskeletal extremity pain in Danish school children – how often and for how long? The CHAMPS study-DK

**DOI:** 10.1186/s12891-017-1859-8

**Published:** 2017-11-25

**Authors:** Signe Fuglkjær, Jan Hartvigsen, Niels Wedderkopp, Eleanor Boyle, Eva Jespersen, Tina Junge, Lisbeth Runge Larsen, Lise Hestbæk

**Affiliations:** 1 Department of Sports Science and Clinical Biomechanics, Faculty of Health Sciences, University of Southern Denmark, Campusvej 55, 5230 Odense M, Denmark; 2 Nordic Institute of Chiropractic and Clinical Biomechanics, Campusvej 55, 5230 Odense M, Denmark; 3 Institute of Regional Health Services Research, University of Southern Denmark, Winsloewparken 193, 5000 Odense C, Denmark; 4 Sports Medicine Clinic, Orthopaedic Department, Hospital Lillebaelt, Østre Hougvej 55, 5500 Middelfart, Denmark; 5 Dalla Lana School of Public Health, University of Toronto, 155 College St, Toronto, ON M5T 3M7 Canada; 6 Department of Rehabilitation, Odense University Hospital, Odense, Denmark and National Centre of Rehabilitation and Palliation, University of Southern Denmark, Odense, Denmark; 7 Health Sciences Research Centre, University College Lillebaelt, Niels Bohrs Allé 1, 5230 Odense M, Denmark; 8 Research and Innovation Center for Human Movement and Learning, Inter-Faculty Educational Resources, University College Lillebælt, Niels Bohrs Alle 1, 5230 Odense M, Denmark

**Keywords:** Adolescent health, Epidemiology, Cohort, Leg, Arm, Limb, Injury, Complaint, Prevalence

## Abstract

**Background:**

Musculoskeletal pain is common in childhood and adolescence, and may be long-lasting and recurrent. Musculoskeletal problems tend to follow adolescents into adulthood, and therefore it is important to design better prevention strategies and early effective treatment. To this end, we need in-depth knowledge about the epidemiology of musculoskeletal extremity problems in this age group, and therefore, the aim of this study was to determine the prevalence, frequency and course of musculoskeletal pain in the upper and lower extremities in a cohort of Danish school children aged 8–14 years at baseline.

**Methods:**

This was a prospective 3-year school-based cohort study, with information about musculoskeletal pain collected in two ways. Parents answered weekly mobile phone text messages about the presence or absence of musculoskeletal pain in their children, and a clinical consultation was performed in a subset of the children.

**Results:**

We found that approximately half the children had lower extremity pain every study year. This pain lasted on average for 8 weeks out of a study year, and the children had on average two and a half episodes per study year. Approximately one quarter of the children had upper extremity pain every study year that lasted on average 3 weeks during a study year, with one and a half episodes being the average. In general, there were more non-traumatic pain episodes compared with traumatic episodes in the lower extremities, whereas the opposite was true in the upper extremities. The most common anatomical pain sites were ‘knee’ and ‘ankle/ft’.

**Conclusion:**

Lower extremity pain among children and adolescents is common, recurrent and most often of non-traumatic origin. Upper extremity pain is less common, with fewer and shorter episodes, and usually with a traumatic onset. Girls more frequently reported upper extremity pain, whereas there was no sex-related difference in the lower extremities. The most frequently reported locations were ‘knee’ and ‘ankle/ft’.

**Electronic supplementary material:**

The online version of this article (10.1186/s12891-017-1859-8) contains supplementary material, which is available to authorized users.

## Background

Musculoskeletal (MSK) pain is common in childhood and adolescence [1–3], and may be long-lasting and recurrent [4–6]. MSK pain in the lower extremities occurs both in children [3, 7] and in adolescents [1], with ankle and foot problems being more common in children [7, 8], and knee problems being more common during adolescence [1]. Importantly, one study found that half the Danish adolescents aged 12–15 years with knee pain also reported knee pain when asked 1 year later [9]. Similarly, in another study, one third of the Finnish children with lower extremity (LE) pain reported its presence one and/or 4 years later [4]. MSK problems tend to follow adolescents into adulthood [10, 11], and therefore, it is important to design better prevention strategies and early effective treatment. To this end, we need in-depth knowledge about the epidemiology of MSK extremity problems in children and adolescents, including frequency and course.

Traditionally, data on MSK health in children and adolescents have been obtained from clinical assessments in emergency departments or in primary care physicians’ practices. This type of research provides valid information about patterns of care-seeking, mainly for traumatic problems such as fractures and distortions [12, 13]. However, such studies rarely provide information about non-traumatic problems, which have been shown to be more common compared to traumatic problems in these age groups [3, 14]. Knowledge about MSK problems in the general population, including non-traumatic problems, has traditionally been collected via questionnaires completed by children or their parents. However depending on recall period, questionnaire data may suffer from recall bias, especially for minor problems [15, 16], and therefore short recall periods are needed to collect reliable estimates [17]. Mobile phone text messages at short time-intervals is one practical and user-friendly method to reduce recall bias [18, 19], and thus, may be a more valid way to track information about MSK problems in children. In addition, this method makes it possible to estimate occurrence by prevalence rather than incidence, which has been suggested to be the most appropriate way to describe non-traumatic MSK problems, because of the long-lasting and recurrent nature of these complaints [20]. Therefore, we set out to determine the frequency and course of upper extremity (UE) and LE pain in 8–14-year-olds. Specifically, we wanted to describe the following four areas using information about their MSK pain reported by their parents via weekly mobile phone text messages, as well as data obtained in a clinical examination for a subset of this cohort:

1. MSK pain as reported by the parents

- ◦The sex-specific prevalence of any type of UE or LE pain
- ◦The frequency and duration of UE or LE pain episodes

2) Pain distribution as reported from the clinical examination

- ◦The prevalence of pain in specific anatomical sites
- ◦The distribution of 1) pain episodes in girls versus boys and 2) traumatic versus non-traumatic episodes including a comparison of traumatic versus non-traumatic episodes in relation to frequency

## Methods

### Setting

This was a prospective three-year school-based cohort study nested within the Childhood Health, Activity and Motor Performance School Study (CHAMPS Study-DK). It started in 2008, and in August 2011, additional funding made it possible to prolong the study until June 2014. It was a dynamic cohort study, thus children could enter or leave the study at any time during the study period. The main purpose of the CHAMPS Study-DK was to evaluate the effectiveness of extra physical education on children’s general health. Schools were divided into two groups: intervention schools received six lessons of physical education per week, whereas control schools only received two lessons. The intervention was performed from 2008 to 2012. With regard to MSK pain, data was obtained over the complete study period (2008 to 2014). The CHAMPS Study-DK is described in detail elsewhere [21]. Data on incidence and prevalence of MSK extremity injuries from 2008 to 2011 have been reported previously [7, 14]. The current study includes data from 2011 to 2014, and the prevalence and course of spinal pain in the children has been reported previously [22].

### Study population

In August 2011, all pupils attending the third to seventh grade in 13 out of 17 public primary schools in the municipality of Svendborg, Denmark, were invited to participate in the study. Svendborg is a Danish municipality with 58,000 inhabitants and is comparable to the rest of Denmark in terms of age, sex and income, but has a slightly higher unemployment rate (5.3% versus 4.5%) [23]. In Svendborg, 84% of children attend public schools. The children and adolescents in the study were from families that represented all levels of socio-economic status.

### Data collection

#### Musculoskeletal pain as reported by the parents

Every Sunday, parents received three mobile phone text message questions (SMS questions); one about presence or absence of MSK pain in their child and two about the child’s sports participation. For this study, only the data in response to the question about MSK pain were used. The exact wording of the question was as follows: “Has [name of child] had any pain during the past week in: 1-Neck or back; 2-Shoulder, arm or hand; 3-Hip, leg or foot; or 4-No pain, and no further instructions were given regarding pain registration. It was possible to report pain in more than one area. If parents did not reply they received up to two reminders at intervals of 48 h. The SMS questions were sent out every week except for 6 weeks during the summer holidays (July and August) and 1 week during the Christmas holidays.

If parents texted a ‘1’, ‘2’ and/or ‘3’ for the MSK pain question, they were telephoned within 5 days by a member of the clinical team, consisting of licenced and experienced physiotherapists and chiropractors. A standardized interview was performed about the nature of their child’s pain, including information about duration of pain and mode of onset. If the pain seemed to be of MSK origin, a clinical examination was scheduled at the child’s school within 2 weeks from the date parents received the SMS question. On the other hand, if the pain was perceived to be non-MSK in nature or had disappeared, no further action was taken. To assemble comprehensive information on all MSK problems, information obtained from the telephone interview or the clinical examination about children being examined or treated elsewhere (e.g. emergency department) was collected concurrently. Relevant information from medical records was registered for analyses without a clinical examination performed by a member of the clinical team.

#### Pain distribution as reported from the clinical examination

A member of the clinical team performed the clinical examination, and parents were informed about the result of the examination by telephone or letter. If deemed necessary, the child was referred to a medical specialist for further examination.

Based on the clinical examination, the UE and LE pain were categorized into one of the following anatomical sites: ‘shoulder’, ‘upper arm’, ‘elbow’, ‘lower arm’, ‘wrist/hand/fingers’, ‘hip/groin’, ‘thigh’, ‘knee’, ‘lower leg’, ‘ankle/ft’, ‘unspecific upper extremity’ and ‘unspecific lower extremity’. If a child had several MSK problems at different anatomical sites within the same extremity, the clinician defined the primary pain site based on the child’s report of impact, and this was used for analyses. Further, for the analyses in the current study, we classified all examined anatomical episodes as either traumatic or non-traumatic using information from the clinical examination. A traumatic episode was defined as an injury resulting from a specific identifiable event, whereas a non-traumatic episode was not related to an identifiable event [24].

### Variables

Descriptive variables, which included age and sex, and outcome variables, are listed below:

#### MSK pain as reported by the parents

- ◦ UE pain the last week (Y/N)
- ◦ LE pain the last week (Y/N)

#### Pain distribution as reported from the clinical examination

- ◦ Anatomical pain site
  - ◦ Shoulder
  - ◦ Upper Arm
  - ◦ Elbow
  - ◦ Lower Arm
  - ◦ Wrist/Hand/Fingers
  - ◦ Hip/groin
  - ◦ Thigh
  - ◦ Knee
  - ◦ Lower leg
  - ◦ Ankle/Foot
  - ◦ Unspecific Upper Extremity
  - ◦ Unspecific Lower Extremity

Anatomical pain sites were divided according to causation

- ◦ Traumatic episodes
- ◦ Non-traumatic episodes

### Data analysis

STATA 15.0 (StataCorp, College Station, Texas, USA) was used for the analyses. UE and LE pain were reported separately, and the significance level was set at 0.05. To avoid breaks in data continuity due to the summer holidays, we chose to report by study year rather than for 3 full calendar years. Therefore, study year 1 represents the period from August 2011 to June 2012 (44 weeks), study year 2 was the period from August 2012 to June 2013 (47 weeks); and study year 3 covers August 2013 to June 2014 (46 weeks).

To obtain a satisfactory observation period, the child had to participate at the start and at the end of a study year, to be included in the analyses. More specifically, children should be included in the study for at least a full study year minus 1 week, e.g. 43 weeks in study year 1. Within this period, missing answers were allowed; however, parents had to respond in at least 85% of the weeks within a study year, or the child would be excluded due to low SMS compliance.

Chi-squared or unpaired t-tests were performed to determine whether there were any differences in demographics between the children who were included in the analysis and those who were not, either due to declining participation in the project, low SMS-compliance, or dropping out of the study.

Research has shown that MSK pain varies between the sexes [1, 25]. Therefore, we calculated sex-specific *prevalence rates* with 95% confidence intervals of UE pain and LE pain for each study year. Trend in prevalence rates with age was assessed using generalized estimating equations.

The *total number of pain weeks* was calculated as the sum of weeks where pain was reported for each study year. They were categorized based on the distribution of data, and results were expressed in numbers, proportions and means with 95% confidence intervals, and medians with interquartile ranges. An episode was deemed to have started when pain was reported in an area from which no pain had been reported in the previous week. Similarly, an episode was deemed to have ended when pain was not reported in the area for at least 1 week. To assess the robustness of this definition, we repeated the analysis using a 4-week ‘no-pain-in-the-area’ gap instead of the 1-week gap. In the 4-week definition, at least 4 weeks of no pain was needed before a subsequent episode was categorized as a *new* episode.

The *number of episodes* per child was calculated as the sum of episodes for each child during a study year.

For each episode, we calculated the *length of an episode* by summing the number of weeks where pain was reported. Number of episodes and length of episodes were categorized based on the distribution of data, and results were expressed in numbers, proportions and means with 95% confidence intervals, and medians with interquartile ranges.

We formulated the following decision rules to account for missing SMS answers. If four or fewer consecutive SMS answers were missing, they were imputed with the same value as the previous week’s SMS answer, provided it was the same as for the week after the missing SMS answers. Otherwise, we defined the end of that episode as occurring at the week prior to the missing SMS answers. If the number of consecutive missing SMS answer weeks was greater than four, we also defined the end of the episode as occurring at the week prior to the missing SMS answer.

We performed a sensitivity analysis to estimate the impact of these decision rules by treating missing answers in two extreme ways to determine the range within which the correct value would lie: first, we imputed the missing answers to be the same as the last SMS answer, regardless of the value of the next report. This would potentially inflate episode lengths and diminish the number of episodes. Second, we imputed the SMS answer ‘no pain’ for all weeks with missing answers, which would do the opposite.

Prevalence rates of pain from each anatomical site based on the clinical examination were calculated with 95% confidence intervals for each study year in the same way as above. Likewise, trend in prevalence rates with age was assessed using generalized estimating equations. To identify possible differences between boys and girls, and between traumatic and non-traumatic episodes regarding anatomical pain sites, results for all 3 study years were used, and the boy:girl ratios and the traumatic:non-traumatic ratios were calculated. Finally, the mean number of episodes per child was calculated for traumatic and non-traumatic pain episodes and a potential difference was tested using a mixed effect regression model, reported by a *p* value.

## Results

### Study sample

**Fig. 1:**
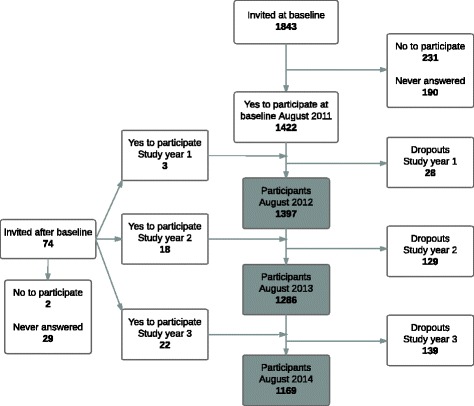
Overview of the participant flow in a cohort of school children (CHAMPS Study-DK; *n* = 1465)

During the entire study period, 1917 children were invited to participate in the CHAMPS-Study DK, and 1465 (76%) were enrolled. During the study period, 296 children dropped out (Fig. 1).

**Fig. 2:**
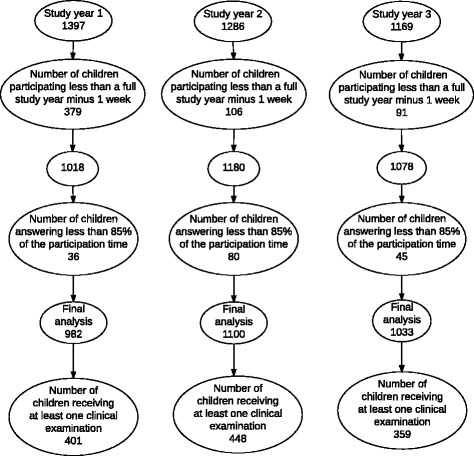
Overview of the exclusion procedure for children in the final analyses

The average weekly response rate for all three study years was 96.4%. After excluding children with low SMS compliance, the final sample consisted of 982 children in study year 1; 1100 children in study year 2; and 1033 children in study year 3. In total, 401, 448 and 359 children received a clinical examination the three study years respectively (Fig. 2)*.* In study year 1, 70 (7.1%) children had an anatomical pain site registered in UEs, and 361 (36.8%) children in the LEs. In study year 2, there were 108 (9.8%) and 392 (35.6%) children with pain in UEs and LEs, respectively, and for study year 3, the numbers were 91 (8.8%) and 312 (30.1%).

The children were 8–14 years of age at baseline in 2011, and mean age increased from 10.7 (SD 1.4) years in study year 1 to 11.6 (SD 1.4) years in study year 2, and 12.5 (SD 1.4) years in study year 3. During all 3 study years, 52% of the children were girls.

We found no significant differences between the children who either declined participation, had low SMS compliance or dropped out, when compared to the study sample in relation to sex, but the dropouts were on average older compared to children who remained in the study (12.5 years of age versus 10.6 years of age, *p* < 0.001).

1. Musculoskeletal pain as reported by the parents

### Upper extremity

#### Sex-specific and age-specific prevalence rates by study year

**Fig. 3:**
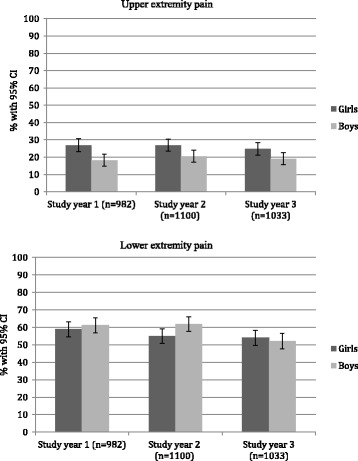
Prevalence rates with 95% confidence intervals of upper extremity and lower extremity pain by study year, from a cohort of Danish school children. CI: confidence intervals

**Fig. 4:**
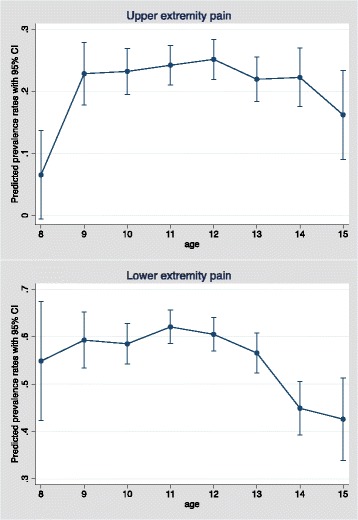
Predicted prevalence rates with 95% confidence intervals of upper and lower extremity pain by age, from a cohort of Danish school children. CI: confidence intervals

Approximately a quarter of the girls and a fifth of the boys reported pain in the UEs, with no change with age (Figs. 3 and 4).

#### Total number of pain weeks

On average, the children who reported UE pain reported its presence close to 3 weeks in study year 1, and this increased to more than 4 weeks in study year 3 (Table 1). In study year 1, 48% of the children reporting UE pain, reported its presence for only 1 week; and in study year 3 this decreased to 40%. Approximately 7% reported pain for 8 weeks or more in study year 1, increasing to 15% in study year 3.

**Table 1:** Proportion of children who experienced upper extremity pain expressed by number of pain weeks, from a cohort of Danish school children

Number of pain weeks	Study year 1 (44 weeks)		Study year 2 (47 weeks)		Study year 3 (46 weeks)	
	n	% (95% CI)	n	% (95% CI)	n	% (95% CI)
1	108	48.7 (41.9–55.4)	116	44.3 (38.2–50.5)	91	40.1 (33.7–46.8)
2	36	16.2 (11.6–21.)	47	17.9 (13.5–23.1)	28	12.3 (8.4–17.3)
3	22	9.9 (6.3–14.6)	29	11.1 (7.5–15.5)	32	14.1 (9.8–19.3)
4	13	5.9 (3.2–9.8)	21	8.0 (5.0–12.0)	22	9.7 (6.2–14.3)
5	11	5.0 (2.5–8.7)	6	2.3 (0.8–4.9)	8	3.5 (1.5–6.8)
6	8	3.6 (1.6–7.0)	6	2.3 (0.8–3.9)	8	3.5 (1.5–6.8)
7	9	4.1 (1.9–7.6)	9	3.4 (1.6–6.4)	4	1.8 (0.5–4.5)
8–43	15	6.8 (3.8–10.9)	28	10.7 (7.2–15.1)	34	15.0 (10.6–20.3)
Total^a^	222	100.0	262	100.0	227	100.0
Median						
(25%–75%)^b^		2 (1–4)		2 (1–4)		2 (1–4)
Mean (95% CI)^b^		2.9 (2.5–3.3)		3.4 (2.9–3.9)		4.4 (3.6–5.2)

children without reported pain are not included

*CI* Confidence intervals

^a^number of participants reporting upper extremity pain during 1 study year

^b^number of weeks

#### Number of episodes

**Fig. 5:**
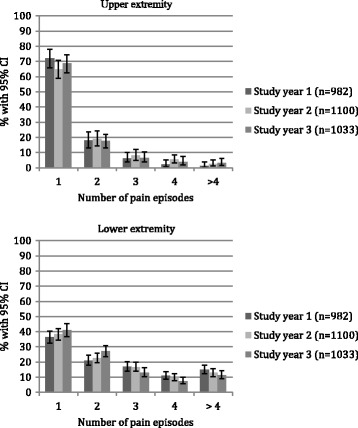
Proportions of children who experienced 1 to more than 4 episodes of upper and lower extremity pain, in a cohort of Danish school children. Children with no reported pain episodes are not included. CI: confidence intervals

Children with UE pain reported on average 1.5 (95% CI 1.3–1.6), 1.6 (95% CI 1.5–1.8) and 1.6 (95% CI 1.4–1.7) episodes per year, for the 3 study years respectively. The median number of episodes was 1 (25%–75%: 1–2), for all 3 study years. Approximately two-thirds of the children with reported UE pain, reported one episode of UE pain per study year, and few children reported more than four episodes per study year (Fig. 5).

#### Length of episodes

The total number of UE pain episodes per study year was 325, 427 and 354, respectively. On average, an episode of UE pain lasted for 2 weeks in study year 1, increasing to almost 3 weeks in study year 3. More than half the episodes lasted for just 1 week, and only a few episodes lasted for more than 12 weeks (Table 2).

**Table 2:** Proportion of episodes according to length of episodes of upper and lower extremity pain, from a cohort of Danish school-children

	Length of episodes	Study year 1 (44 weeks)		Study year 2 (47 weeks)		Study year 3 (46 weeks)	
Upper extremity		n	% (95% CI)	N	% (95% CI)	N	% (95% CI)
	1 week	197	60.6 (55.1–65.9)	283	66.3 (61.6–70.6)	180	50.9 (45.5–56.2)
	2–3 weeks	86	26.5 (21.7–31.6)	86	20.1 (16.4–24.3)	107	30.2 (25.5–35.3)
	4–11 weeks	40	12.3 (8.9–16.4)	55	12.9 (9.9–16.4)	55	15.5 (11.9–19.7)
	≥ 12 weeks	2	0.6 (0.1–2.2)	3	0.7 (0.1–2.0)	12	3.4 (1.8–5.8)
	Total^a^	325	100.0	427	100.00	354	100.0
	Median						
	(25%–75%)		1 (1–2)		1 (1–2)		1 (1–3)
	Mean (95% CI)		2.0 (1.8–2.2)		2.1 (1.9–2.3)		2.8 (2.4–3.1)
Lower extremity	1 week	814	51.3 (48.8–53.8)	816	50.9 (48.4–53.4)	615	48.0 (45.3–50.8)
	2–3 weeks	403	25.4 (23.3–27.6)	407	25.4 (23.3–27.6)	337	26.3 (23.9–28.8)
	4–11 weeks	316	19.9 (18.0–22.0)	297	18.5 (16.7–20.5)	247	19.3 (17.2–21.6)
	≥ 12 weeks	54	3.4 (2.6–4.4)	83	5.2 (4.1–6.4)	81	6.3 (5.1–7.8)
	Total^a^	1587	100.0	1603	100.0	1220	100.0
	Median						
	(25%–75%)		1 (1–3)		1 (1–3)		2 (1–4)
	Mean (95% CI)		3.0 (2.8–3.2)		3.3 (3.1–3.5)		3.6(3.4–3.9)

children without reported pain are not included

*CI* Confidence interval^a^number of pain episodes during 1 study year

### Lower extremity

#### Prevalence by study year

Approximately half the children reported LE pain, with no difference between the sexes (Fig. 3). The risk of reporting LE pain decreased with age, odds ratio 0.91 (*p* value = 0.001). More specifically, there was a statistically significant decrease in prevalence rate from the age of 11 to 15 years (Fig. 4).

#### Total number of pain weeks

During all 3 study years, approximately 20% of the children reporting LE pain did so for 1 week, and another 20% reported LE pain for 12 weeks or more. The average total number of pain weeks was 8 for each study year (Table 3).

**Table 3:** Proportion of children who experienced lower extremity pain by number of pain weeks reported for each study year, from a cohort of Danish school children

Number of pain weeks	Study year 1 (44 weeks)		Study year 2 (47 weeks)		Study year 3 (46 weeks)	
	n	% (95% CI)	n	% (95% CI)	n	% (95% CI)
1	118	20.0 (16.8–23.5)	140	21.8 (18.2–25.2)	120	21.9 (18.5–25.6)
2	83	14.1 (11.4–17.1)	77	12.0 (9.6–14.8)	77	14.0 (11.2–17.2)
3	57	9.7 (7.4–12.3)	74	11.5 (9.2–14.3)	48	8.7 (6.5–11.4)
4	40	6.8 (4.9–9.1)	58	9.1 (6.9–11.5)	41	7.5 (5.4–10.0)
5	38	6.4 (4.6–8.7)	28	4.4 (2.9–6.3)	29	5.3 (3.6–7.5)
6	25	4.2 (2.8–6.2)	20	3.1 (1.9–4.8)	29	5.3 (3.6–7.5)
7	30	5.1 (3.5–7.2)	27	4.2 (2.8–6.1)	17	3.1 (1.81–4.9)
8	16	2.7 (1.6–4.4)	22	3.4 (2.2–5.2)	21	3.8 (2.4–5.8)
9	17	2.9 (1.7–4.6)	19	3.0 (1.8–4.6)	16	2.9 (1.7–4.7)
10	14	2.4 (1.3–3.9)	13	2.0 (1.1–3.4)	15	2.7 (1.5–4.5)
11	17	2.9 (1.7–4.6)	12	1.9 (1.0–3.2)	9	1.6 (0.8–3.1)
12	12	2.0(1.1–3.5)	14	2.2 (1.2–3.6)	9	1.6 (0.8–3.1)
13–45	123	20.9 (17.6–24.4)	137	21.4 (18.3–24.8)	118	21.5 (18.1–25.2)
Total^a^	590	100.0	641	100.0	549	100.00
Median						
(25%–75%)^b^		4(2-11)		4 (2–11)		4 (2–10)
Mean (95% CI)^b^		8.3 (7.5–9.1)		8.3 (7.5–9.1)		8.7 (7.8–9.6)

children without reported pain are not included

*CI* Confidence intervals

^a^number of participants reporting lower extremity pain during 1 study year

^b^number of weeks

#### Number of episodes

Children with LE pain reported on average 2.7 (95% CI 2.5–2.9), 2.5 (95% CI 2.4–2.7) and 2.4 (95% CI 2.2–2.5) episodes per study year, for the 3 years respectively. Approximately one-third of the children reported one episode per study year, and one-third reported three episodes or more per study year (Fig. 5).

#### Length of episodes

The total number of LE pain episodes was 1587, 1603 and 1220, for the 3 study years respectively. An episode of LE pain lasted on average 3 weeks in study year 1, increasing to 3.5 weeks in study year 3. Approximately half the episodes lasted for 1 week in all 3 study years. In study year 1, 3% of the episodes lasted more than 12 weeks, and in study year 3, it was 6% (Table 2).

### Pain distribution as reported from the clinical examination

#### Prevalence of anatomical pain sites by study year

The subset of the cohort with registration of an anatomical pain site consisted approximately of one-third of the children with reported UE pain, and close to two-thirds of those with reported LE pain. In total, 1729 anatomical pain sites were registered during the three study years.

**Fig. 6:**
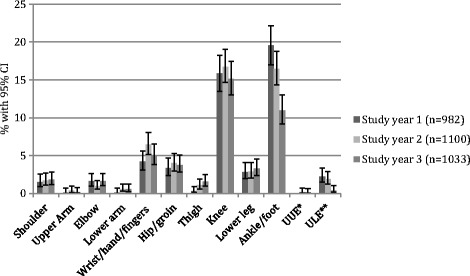
Prevalence of anatomical pain sites by study year, obtained through clinical examination from a cohort of Danish school children. *unspecific upper extremity, **unspecific lower extremity

The two most frequent pain sites were ‘knee’ and ‘ankle/ft’. ‘Knee’ was registered in approximately 15% of the children each study year. ‘Ankle/ft’ was registered in 19% of the children in study year 1, decreasing to 10% in study year 3. The least frequent pain sites were upper and lower arm, with prevalence rates of less than 1% (Fig. 6). The risk of reporting ‘ankle/ft’ decreased with age, odds ratio 0.82 (*p* value <0.001). More specifically, there was a statistically significant decrease in prevalence rate from the age of 10 to 14 years.

#### Distribution by sex

**Fig. 7:**
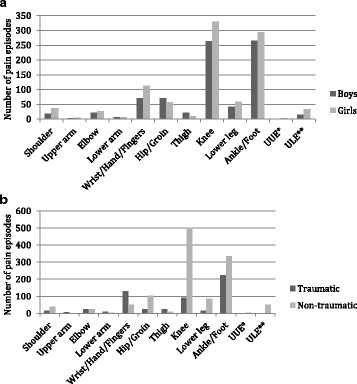
Total number of pain episodes over three study years by **a**) sex and **b**) causation, in a subset of a cohort of Danish school children. The total number of pain episodes was 1729, distributed in 777 children. * unspecific upper extremity ** unspecific lower extremity

In general, more pain sites were registered in girls than boys, noticeably ‘unspecific lower extremities’ 2.3 times, ‘shoulder’ 2.1 times and ‘wrist/hand/fingers‘ 1.6 times more. Only ‘thigh’ pain was more frequent in boys, with 2.2 times more pain episodes than in girls. In ‘ankle/ft’, ‘hip/groin’, ‘lower arm’ and ‘elbow’, there was an equal distribution between boys and girls (Fig. 7a).

#### Distribution by complaint type

In general, for the UEs, there were more traumatic than non-traumatic episodes. For ‘wrist/hand/fingers’, ‘upper arm’ and ‘lower arm’, there were 2.5 to 6.0 times more traumatic episodes compared with non-traumatic episodes, whereas there was an equal distribution between traumatic and non-traumatic episodes in the ‘elbow’. The opposite pattern was found in ‘shoulder‘, with 2.7 times more non-traumatic episodes compared with traumatic. Conversely, in the LEs, non-traumatic episodes were more common than traumatic episodes. In ‘knee’, ‘hip/groin’ and ‘lower leg’ 4.0 to 6.1 times more episodes were categorized as non-traumatic, and in ‘ankle/ft’ there were approximately 1.5 times more non-traumatic episodes. The opposite pattern was found in ‘thigh’ with 3.0 times more episodes categorized as traumatic (Fig. 7b). The prevalence rates of non-traumatic ‘ankle/ft’ episodes decreased significantly from 13% (95% CI 11.0–15.3) in study year 1 to 6% (95% CI 4.6–7.6) in study year 3.

#### Comparison of traumatic versus non-traumatic pain episodes by number and length of episodes

On average, children with non-traumatic episodes reported more episodes compared with children with traumatic episodes, 1.8 (95% CI 1.7–1.9) versus 1.5 (95% CI 1.4–1.5) (*p* value <0.001).

#### Definition of a new episode (sensitivity analysis)

We compared the definition of an episode used in the analyses (a new episode was deemed to have started when pain was reported in an area from which no pain was reported the previous week) to a four-week ‘no-pain-in-the-area’ gap. As expected, the mean number of episodes decreased whereas the mean length of episodes increased, when 4 weeks of ‘no pain’ were needed before a subsequent episode was considered to be a new episode. Importantly, analyses showed the same pattern of MSK pain with more LE pain and more LE episodes compared with UE episodes. The length of episodes increased on average with 70% in LE, and 40% in UE when a four-week-gap of ‘no pain’ was needed, indicating LE episodes were more recurrent compared with UE episodes (Additional file 1).

#### Missing data

For UEs, both when missing data were imputed as the same as the last answer and as no pain, we found similar results. For LEs, analyses resulted in fewer and longer episodes when missing data were imputed as the same as the last SMS answer. Furthermore, we found shorter pain periods in two out of three situations, when missing data were imputed as ‘no pain’ (Additional file 2).

## Discussion

In a three-year study of Danish school children aged 8–14 years at baseline, we found that approximately half the children had LE pain, and one-quarter of the children reported UE pain every study year. The children with LE pain had on average more and longer pain episodes compared with the children with UE pain. The two most common anatomical pain sites for the subset of the children, who were examined, were ‘knee’ and ‘ankle/ft’.

Previous studies have also found LE pain to be more common than UE pain [25–28], but to our knowledge, this is the first study to closely follow MSK problems in this age group over a longer period of time. Although the exact location and type of problem is unknown, the number of episodes of LE pain demonstrates that more than half of the affected children have recurrent problems in the LE. This confirms results from two other cohort studies [4, 9]. Our finding of ‘knee’ and ‘ankle/ft’ as the most common pain sites is also in line with previous findings [1, 8]. In the years leading up to this study, information about MSK pain was collected from the same cohort, where ‘ankle/ft’ injuries were found to be the most common, followed by ‘knee’ injuries [7]. We found a significant decrease in the prevalence rate of ‘ankle/ft’ problems with age, especially in non-traumatic pain episodes. This may be because of a reduction in growth-related symptoms, or potentially a decreased amount of physical activity in adolescence.

### Methodological considerations

There was not a large attrition bias, but the dropouts were slightly older than the children who remained in the study, which potentially could result in an underestimation of pain, since MSK pain seems to increase during adolescence [26, 29, 30].

There might be some limitations with regard to the SMS answers. Parents answered SMS questions continuously every week for more than 5 years, and therefore some response fatigue could be anticipated. Nevertheless, the response rate was high, although we do not know if some parents reported ‘no pain’ to avoid a subsequent phone call, again resulting in an underestimation of the reporting of pain. Furthermore, the breaks in data continuity were also a limitation, as we do not know how the holidays influence the prevalence of MSK pain.

Parent reporting is often used as a proxy measure for children reporting their pain themselves. To determine concordance between parent reporting and the children’s actual pain, 685 children, aged 8 to 14 years, were questioned about presence, location and severity of MSK pain. When compared with the SMS answers, poor parent-child concordance was found [31]. Interestingly, the child often reported pain that was not reported by their parents, whereas the opposite was rarely the case. Thus, we probably have lower estimates of pain in this study, compared with child-reported data. A better concordance was seen for pain of greater intensity, which could indicate that parents did not report minor pain. This is in line with another study showing that minor complaints were more likely to be under-reported by parents [16], whereas better concordance was found when children were more severely ill [32].

Another limitation may be an unknown change in the parent/child relationship with age. We expect this potential bias to be limited, because of the children’s relatively young age. If such a bias was present, it was most likely to be independent of location, i.e. the effect would be the same for all pain sites. It might be that non-traumatic episodes are easier to hide, potentially resulting in a higher traumatic/non-traumatic ratio with age, if the child became less communicative.

Major strengths include the large population-based cohort, and the high response rate of the text messages. Furthermore, our results appear robust because imputing missing data and sensitivity analyses did not change our results. We believe that the combination of these parental pain reports, and the information from clinical examinations, provides a comprehensive overview of MSK problems in this age group, but more research in other settings of general and clinical populations is needed.

## Conclusion

Lower extremity pain among children and adolescents is common, recurrent and most often of non-traumatic origin. Upper extremity pain is less common, with fewer and shorter episodes, and usually with a traumatic onset. Girls more frequently reported upper extremity pain, whereas there was no sex-related difference in the lower extremities. The most frequently reported locations were ‘knee’ and ‘ankle/ft’. These findings should encourage a stronger focus on prevention and early effective treatment of lower extremity pain in children and adolescents.

## Additional files

Additional file 1: Pain episodes. Definition of a pain episode. (DOCX 78 kb)

Additional file 2: Missing data. Decision rules in imputation of missing data (DOCX 94 kb)

## Abbreviations

CHAMPS Study-DK: Childhood Health, Activity and Motor Performance School Study
LE: Lower extremity
MSK: Musculoskeletal
SMS answer: Mobile phone text message answer
SMS question: Mobile phone text message question
UE: Upper extremity
