# Supplementary material for: Musculoskeletal extremity pain in Danish school children – how often and for how long? The CHAMPS study-DK
Source: BMC Musculoskelet Disord. 2017 Nov 25;18:492. doi: 10.1186/s12891-017-1859-8 (PMC5702201; doi:10.1186/s12891-017-1859-8)
Supplement: Supplementary file 1 — Pain episodes. Definition of a pain episode. (DOCX 78 kb) [file 12891_2017_1859_MOESM1_ESM.docx]

**Additional file 1**

**Definition of a pain episode**

*One week* (definition used in the analyses): A pain episode was deemed to have started when pain was reported in an area from which no pain had been reported in the previous week. Similarly, an episode was deemed to have ended when pain was not reported in the area for at least 1 week.

*Four weeks*: Here, at least 4 weeks of no pain was needed before a subsequent episode was categorized as a new episode. Therefore, a pain episode was deemed to have started when pain was reported in an area, from which no pain had been reported in at least the previous 4 weeks.

| Box A |
| --- |
| 0 3 3 3 3 0 0 3 3 3 0 |
| 3: lower extremity pain  0: no pain |

Therefore, in box A, the ‘1 week’ definition would result in two pain episodes of 4 and 3 weeks respectively, whereas the ‘4 weeks’ definition would result in one pain episode of 9 weeks.

To assess the robustness of this definition used in the analyses, we repeated the analysis using the ‘4 weeks’ definition. Comparisons of the two definitions are seen below.

| Comparison of two definitions of a new episode in relation to number and length of episodes  (n = number or length of episodes) | | | | | | | |
| --- | --- | --- | --- | --- | --- | --- | --- |
|  | **Study year 1** | | **Study year 2** | | | **Study year 3** | |
|  | 1 week  n (95% CI) | 4 weeks  n (95% CI) | | 1 week  n (95% CI) | 4 weeks  n (95% CI) | 1 week  n (95% CI) | 4 weeks  n (95% CI) |
| **Upper extremity** | | | | | | | |
| Mean number of episodes per child | 1.46  (1.33-1.60) | 1.27  (1.18-1.36) | | 1.64  (1.50-1.77) | 1.41  (1.31-1.51) | 1.57  (1.43-1.72) | 1.30  (1.19-1.40) |
| Mean length of episodes in weeks | 1.96  (1.75-2.16) | 2.75  (2.37-3.14) | | 2.08  (1.87-2.29) | 2.89  (2.47-3.30) | 2.77  (2.40-3.14) | 3.91  (3.26-4.57) |
| **Lower extremity** | | | | | | | |
| Mean number of episodes per child | 2.71  (2.55-2.87) | 1.87  (1.78-1.97) | | 2.52  (2.38-2.66) | 1.83  (1.74-1.93) | 2.35  (2.21-2.50) | 1.70  (1.60-1.79) |
| Mean length of episodes in weeks | 3.02  (2.84-3.20) | 5.59  (5.10-6.07) | | 3.30  (3.08-3.51) | 5.59  (5.11-6.08) | 3.62  (3.36-3.89) | 6.12  (5.51-6.72) |
| *1 week:* Defined as a report of pain followed by at least 1 week without pain from the same area. The end of an episode occurred when pain was not reported in the area for at least 1 week.  *Up to 4 weeks: at least* 4 weeks of ’no pain’ was needed before a subsequent episode was considered to be a new episode  CI: confidence interval | | | | | | | |
