# Supplementary material for: Musculoskeletal extremity pain in Danish school children – how often and for how long? The CHAMPS study-DK
Source: BMC Musculoskelet Disord. 2017 Nov 25;18:492. doi: 10.1186/s12891-017-1859-8 (PMC5702201; doi:10.1186/s12891-017-1859-8)
Supplement: Supplementary file 2 — Missing data. Decision rules in imputation of missing data (DOCX 94 kb) [file 12891_2017_1859_MOESM2_ESM.docx]

| **Additional file 2** Sensitivity analyses of missing data. Data were analysed in two different ways | | | | | | | | | | | | | | |
| --- | --- | --- | --- | --- | --- | --- | --- | --- | --- | --- | --- | --- | --- | --- |
|  | Primary data | | | | | Missing imputed as the same as last answer (+pain) | | | | Missing imputed as ’4’ (no pain) | | | | |
|  | Number of episodes | | Length of episodes | | | Number of episodes | | Length of episodes | | Number of episodes | | | Length of episodes | |
|  | Mean  (95% CI) | Median  (25%-75%) | Mean  (95% CI) | Median (25%-75%) | | Mean  (95% CI) | Median (25%-75%) | Mean  (95% CI) | Median  (25%-75%) | Mean  (95% CI) | | Median (25%-75%) | Mean  (95% CI) | Median (25%-75%) |
| **Upper extremity** | | | | |  | | | | | |  | | | |
| Study year 1 | 1.46  (1.33-1.60) | 1  (1-2) | 1.96  (1.75-2.16) | 1  (1-2) | | 1.51  (1.40-1.62) | 1  (1-2) | 2.10  (1.88-2.33) | 1  (1-2) | 1.58  (1.46-1.71) | | 1  (1-2) | 1.96  (1.75-2.16) | 1  (1-2) |
| Study year 2 | 1.64  (1.50-1.77) | 1  (1-2) | 2.08  (1.87-2.29) | 1  (1-2) | | 1.62  (1.53-1.72) | 1  (1-2) | 2.22  (1.96-2.47) | 1  (1-2) | 1.69  (1.58-1.78) | | 1  (1-2) | 2.08  (1.86-2.29) | 1  (1-2) |
| Study  year 3 | 1.57  (1.43-1.72) | 1  (1-2) | 2.77  (2.40-3.14) | 1  (1-3) | | 1.62  (1.50-1.74) | 1  (1-2) | 2.98  (2.52-3.43) | 2  (1-3) | 1.66  (1.55-1.78) | | 1  (1-2) | 2.77  (2.40-3.14) | 1  (1-3) |
| **Lower extremity** | | | | | | | | | | | | | | |
| Study  year 1 | 2.71  (2.55-2.87) | 2  (1-4) | 3.02  (2.84-3.20) | 1  (1-3) | | **2.50**  (2.41-2.59) | 2  (1-3) | **3.28**  (3.05-3.52) | 2  (1-3) | 2.59  **(2.50-2.68)** | | 2  (1-3) | 3.0  (2.84-3.20) | 1  (1-3) |
| Study  year 2 | 2.52  (2.38-2.66) | 2  (1-3) | 3.30  (3.08-3.51) | 1  (1-3) | | **2.32**  (2.24-2.41) | 2  (1-3) | **3.58**  (3.29-3.87) | 2  (1-3) | 2.40  **(2.32-2.48)** | | 2  (1-3) | 3.30  (3.08-3.51) | 1  (1-3) |
| Study  year 3 | 2.35  (2.21-2.50) | 2  (1-3) | 3.62  (3.36-3.89) | 2  (1-4) | | 2.22  **(2.13-2.30)** | 2  (1-3) | **3.96**  (3.59-4.33) | 2  (1-4) | 2.32  (2.23-2.41) | | 2  (1-3) | 3.62  (3.36-3.89) | 2  (1-4) |
| bold: result different compared with results in the primary data  CI: confidence interval | | | | | | | | | | | | | | |

**Additional file 2**

**Missing mobile phone text message answers (SMS answers)**

1. In the analysis described below as ‘primary data’, the missing SMS answers were imputed according to the defined decision rules.

2. In the analysis described below as ‘missing imputed as the same as the last SMS answer (+pain)’, all missing SMS answers were imputed as the same as the last available SMS answer.

3. In the analysis described below as ‘missing imputed as ‘4’ (no pain)’, all missing SMS answers were imputed as ‘no pain’.

A sensitivity analysis was performed to estimate the impact of the decision rules. For scenario two and three, mean and median number of episodes, and mean and median length of episodes were calculated, and results were compared with the primary data.
